# Determination of Hg in Farmed and Wild Atlantic Bluefin Tuna (*Thunnus thynnus* L.) Muscle

**DOI:** 10.3390/molecules24071273

**Published:** 2019-04-01

**Authors:** Anna Annibaldi, Cristina Truzzi, Oliana Carnevali, Paolo Pignalosa, Martina Api, Giuseppe Scarponi, Silvia Illuminati

**Affiliations:** 1 Dipartimento di Scienze della Vita e dell’Ambiente, Università Politecnica delle Marche, Via Brecce Bianche, 60131 Ancona, Italy; o.carnevali@univpm.it (O.C.); m.api@pm.univpm.it (M.A.); g.scarponi@univpm.it (G.S.); s.illuminati@univpm.it (S.I.); 2 Oceansis s.r.l., 80056 Ercolano NA, Italy; oceanissrl@gmail.com

**Keywords:** Atlantic bluefin tuna, Mediterranean Sea, selenium, mercury, atomic absorption spectroscopy, health benefit value

## Abstract

Mercury (Hg) is a well-known toxic element, diffused in the environment, especially in the Mediterranean Sea which is rich in cinnabar deposits. Mercury bioaccumulation in fish is of great concern, especially for top-level aquatic predators (e.g., shark, tuna, swordfish) and above all for species of large human consumption and high nutritional value. This work aimed to determine Hg concentrations in farmed and wild Atlantic Bluefin tuna (*Thunnus thynnus*) caught in the Mediterranean area in order to evaluate the level of Hg bioaccumulation. selenium (Se) content was also determined, since this element is an antagonist of mercury toxicity. Mercury and Se were analysed by atomic absorption spectrometry after microwave digestion of the samples. Hg content in farmed tuna was below the legal limit (1 mg/kg, wet weight, w.w.) for all specimens (0.6 ± 0.2 mg/kg), whereas the wild ones had a content over the limit (1.7 ± 0.6 mg/kg); Se concentration was higher in farmed specimens (1.1 ± 0.9 mg/kg) compared to wild ones (0.6 ± 0.3 mg/kg). A safe seafood could show a Se/Hg ratio >1 and a health benefit value (HBV_Se_) > 0: farmed tuna had higher values than the wild specimens (Se/Hg 5.48 vs. 1.32; HBV_Se_ 11.16 vs. 0.29). These results demonstrate that for Hg, there is a better risk/benefit ratio in farmed *T. thynnus*. making it safer than wild tuna.

## 1. Introduction

Mercury (Hg) is a highly toxic element of natural and anthropogenic origin. Mercury accumulation in the environment is a worldwide and recognized problem, since it can be found in the atmosphere, biosphere and geosphere [1]. In aquatic environments, mercury is readily transformed by chemical and biological (i.e., bacterially mediated) pathways into organomercury compounds such as methylmercury (MeHg), greatly affecting its solubility, volatility, bioavailability and toxicity [2]. Methylmercury can be bioaccumulated more efficiently than other trace metals along the food chain; it represents the most toxic mercury species and is known to have numerous adverse effects, including neurotoxicity, genotoxicity and endocrine disruption on a wide range of vertebrates, including fish [3,4], and invertebrate species [5,6,7,8]. Many studies [9,10,11,12,13] have shown that most of the mercury in fish tissues is methylmercury (90%) and this percentage does not vary by age of the fish.

The Mediterranean Sea is characterized by the presence of large cinnabar deposits (HgS) [10], accounting for about 65% of the world’s mercury reserves, even if it covers only about 1% of the world’s oceans: hence this area is influenced by mercury contamination both from natural and anthropogenic sources (e.g., use in products and by industry). For these features, this environment is very interesting as bioaccumulation site for biota.

The presence of this metal in commercially important seafood captured in the Mediterranean Sea is of major concern for human healthy. In fact, seafood, especially large predatory pelagic fish, represents the most important environmental source of human mercury exposure [12]; consequently, it is desirable to regulate seafood consumption to minimize the risk of mercury accumulation to toxic levels in consumers, especially in sensitive categories, such as babies and pregnant women. For this purpose, a limit for total Hg concentration was established by Food and Agriculture Organization (FAO) and World Health Organization (WHO) in fish for human consumption (1 mg/kg, wet weight, w.w. for predatory fish and 0.5 mg/kg w.w. for non-predatory species) [14,15].

Tuna is an apex predator fish, so it can bioaccumulate relevant amounts of toxic elements, like Hg. Besides, in the last decades the consumption of seafood, and especially of tuna, has increased [16,17] due to more information about the nutritional benefits of this food and to the general increase in the sushi market. Even if the scientific community has been studying Hg bioaccumulation in tuna since 1970 [18], the debate on the concomitant source is still open: is the level of Hg in organisms a background level, or is it influenced by the environment (emission, anthropogenic inputs, industrial activities, etc.) or by predatory area/diet? In this study, the level of Hg in Mediterranean bluefin tuna, both wild and farmed, were investigated to answer these questions. Possible relations with biometric parameter were also considered. Few studies have been carried out on this species to assess the comparison of mercury content between farmed and wild specimens [19,20] and, to the best of our knowledge, this is the first study on Atlantic bluefin tuna (ABFT).

Since selenium shows protective effects against mercury bioaccumulation, we also determined its concentration in all specimens sampled, to evaluate the differences due to different diets and to assess the risk value for the consumer. The antagonist effect of Se on Hg has been well known since the 1970s [21], when it was suggested that an excess of Se over Hg, computed as a molar ratio of Se:Hg > 1, is more protective than a lower value. Furthermore, recent studies on the toxicological effects on fish [22] have underlined the importance of Se content since Hg toxicity could appear even at levels lower than the legal limit (1 mg/kg). Moreover, the apical species could show a lower Se:Hg ratio, because Se has a homeostatic regulation, whereas Hg content generally increases with size and trophic level. However, Se:Hg ratio does not represent the best parameter to assess the benefit value of fish meat, because the effective quote of free Se, available for physiological functions, is not taken into account.

In fact, the protective action of Se against Hg is related mainly to the formation of Hg-Se complexes [23], but this mechanism in food contaminated by Hg could reduce the Se quota amount necessary for normal body functions. Therefore, a more suitable index, the selenium health benefit value (HBV_Se_) was recently introduced [24] to provide a clear instrument to better understand the quote of relative Se available that remains after its Hg-interaction (defined henceforth as bioavailable in the paper) and to classify the risk posed by consuming these foods. The sign indicates whether the food would improve or diminish the Se status, while the scale of the value proportionately reflects the Se surplus or deficit associated with eating that particular seafood. In this context, this paper is the first report that evaluates the risk/benefit index in ABFT captured in the Mediterraneans Sea using HBV_Se_.

Some authors found that lipid accumulation in tuna appears to have a dilution effect on mercury content associated with fish tissues [25,26]: this result could be particularly important within farmed tuna, as the farming process greatly increases the lipid content of tissues. For this reason, we also determined in this study the lipid content in muscle tissue of farmed and wild tuna.

The objectives of the present study were: (1) to determine mercury levels in farmed and wild ABFT and investigate their relationship with size and gender, (2) to give recommendations regarding the safe consumption of this seafood by evaluating the Se/Hg ratio and the HBV_Se_ index.

## 2. Materials and Methods

### 2.1. Sample Collection and Treatments

The animals were sampled under the guidelines Art 36, par.1 Reg (EU) N° 508/2014 [27]. The procedures did not include animal experimentation, so ethics approval was not necessary in accordance with the Italian legislation.

#### 2.1.1. Sampling

**Figure 1:**
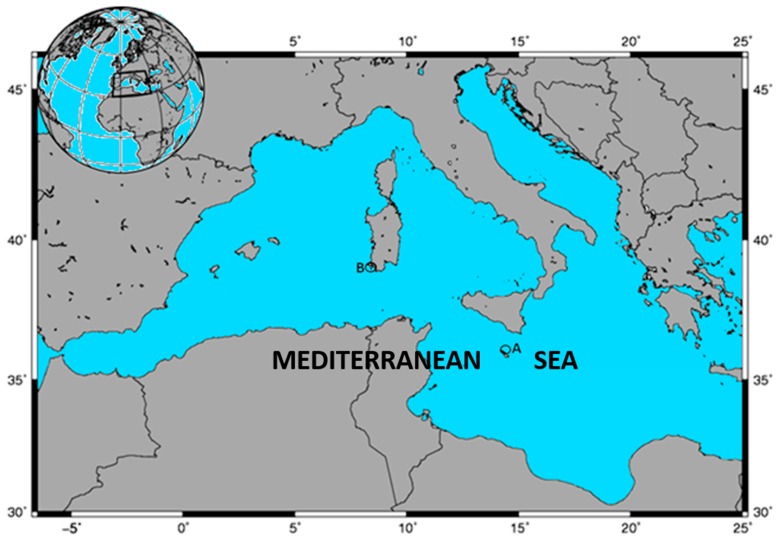
Map of tuna sampling in different areas of the Mediterranean Sea: (**A**), tuna farmed in fish farm Fish and Fish (Malta); (**B**), wild tuna Carloforte tonnare (Carloforte, CA, Sardinia, Italy).

Farmed samples of ABFT *Thunnus thynnus* specimens were caught by purse seine from spawning grounds around the Mediterranean Sea during May–June 2015 (Figure 1) [28].

Fish were immediately moved into towing cages and transported over a period of several months (5–6 months) to the tuna fish farm Fish and Fish Ltd. (Għaxaq, South–East Malta) for fattening. Fish were fed with defrosted raw fish, such as Pacific mackerel *Scomber japonicus*, Atlantic mackerel *Scomber scombrus*, and Atlantic herring *Clupea harengus*.

In total, 40 bluefin tuna specimens (20 males and 20 females) were obtained from the tuna fish farm in November 2015, during the post-spawning period. Curved fork length (from the tip of the upper jaw to the fork of the caudal fin) and total body weight were measured for all tuna fish sampled. The overall mean length was 228 ± 33 cm (males: 240 ± 31 cm, females: 216 ± 31 cm). The overall mean body weight was 238 ± 93 kg (males: 279 ± 86 kg, females: 198 ± 83 kg). Sex of fish was determined by examining gonads under a dissecting microscope. All fish were in the adult stage. Muscle samples were obtained from the upper part of the back, frozen immediately on dry ice, and then stored at −80 °C until analysis. For each specimen, three independent samples of muscle (about 10 g each) were collected.

Wild samples of ABFT were caught by traps around the island of Sardinia, in Carloforte Tonnare during May–June 2017, (Figure 1). In total, 33 bluefin tuna specimens (19 male and 14 female) were sampled. Curved fork length (from the tip of the upper jaw to the fork of the caudal fin) and total body weight were measured for all tuna fish sampled. The overall mean length was 135 ± 23 cm (males: 129 ± 11 cm, females: 133 ± 11 cm). The overall mean body weight was 45 ± 26 kg (males: 40 ± 11 kg, females: 43 ± 7 kg). Even in this case, the sex of fish was determined by examining gonads under a dissecting microscope. Muscle samples were obtained from the dorsal part near the back, frozen immediately on dry ice, and then stored at −80 °C until analysis. For each specimen, three independent samples of muscle (about 10 g each) were collected.

#### 2.1.2. Samples Treatment

For Hg and Se determination, tissues were minced, homogenized (homogenizer MZ 4110, DCG Eltronic), and divided in aliquots of about 0.5 g each. Analyses were carried out on three aliquots per fish. For Hg determination, no other treatments were necessary. For Se analysis, after sample homogenization, we introduced a freeze-drying process that allows a complete loss of water at low temperature (−20 °C) and at low pressure. Tissues were accurately weighed and freeze-dried (Edwards EF4 modulyo, Crawley, Sussex, England) until constant weight (±0.2 mg). Samples were transferred in a Teflon extraction vessel of a microwave accelerated reaction system, MARS-X, 1500 W (CEM, Mathews, NC, USA) and digested without any pre-treatment with a mixture of 3 mL of HNO_3_ and 3 mL of H_2_O_2_. microwave (MW) vessels were 100-mL HP-500 plus in Teflon perfluoroalkoxy copolymer (PFA) from CEM (maximum pressure 350 psig, pounds per square inch gauge, maximum temperature 210 °C). To control temperature and pressure, an HP-500 plus control vessel, filled with the same matrix as that present in the sample vessel, was always used. The system makes it possible to operate in four modalities: standard control, power/time control, ramp to temperature, ramp to pressure. The program used for tissue digestion is reported in Supplementary Table S1 in the Supplementary Material section.

#### 2.1.3. Mercury and Selenium Analysis

The total mercury content was quantified by thermal decomposition amalgamation atomic absorption spectrometry using a direct mercury analyzer (DMA-1, Milestone, Sorisole (BG), Italy). Using this technique, the samples are heated in a quartz container using compressed air (purity of 99.998%) as the oxidant gas. The Hg vapours pass through a catalyst, and the products of combustion are then removed and trapped in a gold amalgamator. High temperatures (850 °C) are applied for desorption and the Hg content is quantified by determining the absorption at 253.7 nm. The optimized conditions for drying and decomposition (pyrolysis) using 60 mg of sample were 200 °C for 120 s and 650 °C for 120 s, respectively. Calibration curve technique was used for the quantification of Hg content. selenium quantitative determinations were carried out with a graphite furnace atomic absorption spectrometry (DUO 240FS AA-GTA120 Graphite Tube Atomizer, Agilent, Santa Clara, CA 95051, USA) equipped with Zeeman background correction. Argon with a purity of 99.998% was used as the carrier gas. Hollow cathode lamp of selenium was used as a light source. Details on the instrumental parameters are reported in the Supplementary Material.

#### 2.1.4. Lipid Determination

Treatments and analysis for lipid determination are explained in detail in a previous work [29]. Homogenized tissues (MZ 4110 homogenizer, DCG Eltronic, Brugherio, Italy) were accurately weighed and freeze-dried (Edwards EF4 modulyo) until constant weight (±0.2 mg). Samples were transferred in a Teflon extraction vessel of a CEM microwave accelerated reaction system, MARS-X, 1500 W with 20 mL of petroleum ether (35 and 60 °C): acetone (2:1, *v*/*v*, Carlo Erba, Milano, Italy), to perform a microwave-assisted extraction (MAE), according to a procedure of Ramalhosa et al. [30]. The extract, filtered through Whatman GF/C filter papers (Ø 90 mm, GE Healthcare Life Sciences, Buckinghamshire, UK) filled with anhydrous sodium sulphate (Carlo Erba) and rinsed twice with further 2 mL of a petroleum ether:acetone mixture, was evaporated under laminar flow inert gas (N_2_) until constant weight. After drying, the mass of extracted lipids was determined by gravimetry.

### 2.2. Laboratory and Apparatus

A clean room laboratory ISO 14644-1 Class 6, with areas at ISO Class 5 under laminar flow, was used for all laboratory activities. The acid-cleaning procedures, used for all the laboratory materials, were performed as described by Illuminati et al., 2014 [31] and Truzzi et al., 2014 [32]. More details are provided in the Supplementary Material section.

### 2.3. Mercury and Selenium Indices

To calculate the risk/benefit value associated with Se and Hg levels in food, two different indices [21,24] were calculated as follows:

Se:Hg molar ratio, from the average Se and average Hg levels in each individual fish (see Tables S3 and S4 in Supplementary Material section). The ratios reported in the paper were calculated from total Se and total Hg:

(1) $${\mathrm{HBV}}_{\mathrm{Se}}=\left(\left[\mathrm{Se}-\mathrm{Hg}\right]/S(1)e\right)\times \left(\mathrm{Se}+\mathrm{Hg}\right)$$

in order to reflect the amount of physiological Se that is in order to reflect the amount of physiological Se that is potentially provided or lost with respect to sequestration by the associated Hg: the relative amount of Se available:

(2) ([Se−Hg]/Se)

is multiplied by the total amount of Hg and Se present in the food (Se + Hg). In this calculation, the concentrations of Hg and Se in mg/kg were used.

### 2.4. Statistical Analysis

Data are expressed as mean ± standard deviation (SD). For each variable, the Student *t*-test was applied to find significant differences between groups at the 95% confidence level [33,34]. Relationships between variables were assessed using Pearson’s correlation coefficient r [35]. All statistical tests were performed with the statistical software STATGRAPHICS (STATGRAPHICS Centurion 2018, Statgraphics Technologies Inc., The Plains, VA, USA).

## 3. Results

All results are expressed in wet weight (w.w). Table 1 and Table 2 show biometric measurements (sex, weight). Results of Hg and Se content in farmed and wild tuna are available in Supplementary Tables S3 and S4.

### 3.1. Accuracy

Quality assurance and quality control were assessed by processing blank samples and certified reference material (dogfish muscle DORM-2, NRCC; Ottawa, ON, Canada). The experimental values obtained for Hg and Se in blanks are negligible compared with the metal content in tuna tissue (<1%). For DORM-2 analysis (*n* = 8), Hg and Se are in agreement with certified values (*p*-values generally >0.05): Hg 4.58 ± 0.10 mg/kg and Se 1.40 ± 0.09 mg/kg against certified mean values Hg 4.43 ± 0.05 mg/kg and Se 1.41 ± 0.02 mg/kg (STATGRAPHICS Centurion 2018).

### 3.2. Mercury and Selenium Content

**Figure 2:**
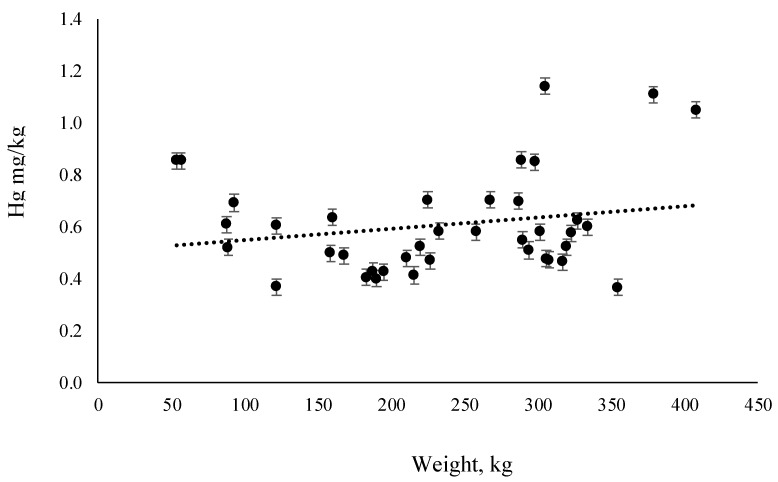
Relationship between Hg concentration (mg/kg w.w.) and body weight (kg) in farmed tuna.

Total concentration of Hg in muscle tissue of farmed tuna is 0.6 ± 0.2 mg/kg. Concerning sex, no differences (*t* = 0.359086, degrees of freedom (Df) = 38, *p* = 0.722, *p* > 0.05) were found in Hg concentration between male and female (0.6 ± 0.2 mg/kg vs. 0.6 ± 0.2 mg/kg respectively). The relationship between Hg concentration and specimen weight was also investigated. Figure 2 shows that Hg concentration increased slightly with tuna weight, but there is no statistical correlation (*p* = 0.214, *p*-value > 0.05).

**Figure 3:**
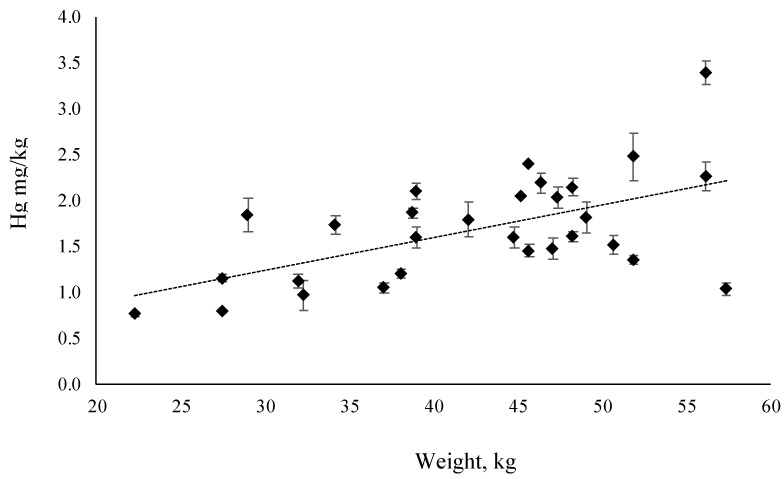
Relationship between Hg concentration (mg/kg w.w.) and body weight (kg) in wild tuna.

Total concentration of Hg in muscle tissue of wild tuna is 1.7±0.2 mg/kg. Even in this case no differences (t = −1.74625, Df = 28, *p* = 0.092, *p* > 0.05) were recorded in Hg concentration between male and female (1.5 ± 0.5 mg/kg vs. 1.9 ± 0.7 mg/kg respectively). Even the relationship between Hg content and specimen weight was investigated: Figure 3 shows that Hg concentration increased linearly with tuna weight, with a statistically significant correlation (*p* = 0.0012).

Se total concentration in farmed tuna was 1.1 ± 0.9 mg/kg. Male and female specimens show no differences in Se content with mean values of 1.3 ± 1.0 mg/kg and 0.9 ± 0.7 mg/kg respectively (*t* = −1.29852, Df = 37, *p* = 0.202). No correlation between Se content and specimen weight was found (*p* = 0.227, *p* > 0.05).

Wild tuna showed a Se total concentration of 0.6 ± 0.3 mg/kg with no statistical differences between male and female specimens (0.7 ± 0.3 mg/kg and 0.6 ± 0.3 mg/kg respectively) (*t* = 0.837801, Df = 30, *p* = 0.408). Even in this case, no correlation between Se content and specimen weight was found (*p* = 0.068, *p* > 0.05).

### 3.3. Lipid Content in Farmed and Wild Specimens

Lipid content of farmed and wild tuna are extensively discussed in a previous work [29]. Briefly, farmed tuna show a higher content of lipids 12 ± 4% compared to wild ones (1.44 ± 1.47%), due probably to their different diets.

## 4. Discussion

### 4.1. Mercury in Farmed and Wild Atlantic BlueFin Tuna (ABFT)

Farmed specimens show Hg levels well below those of wild ones, with a statistically significant difference (*t* = −10.775, Df = 67, *p* = 1.96 × 10^−11^, *p* < 0.0001) between the two groups, even if the body weight of farmed tuna (93–408 kg) (Table 1) is higher than the wild one (28–57 kg) (Table 2). These results are in agreement with the non-linearity of the relationship between Hg content and weight of farmed tuna (Figure 2): similar results were reported by Vizzini et al. [36], who found that farmed tuna had lower Hg levels than wild tuna, with a mean value comparable with our specimens (about 0.5 mg/kg). There are different possible explanations for this result t. The first one is ascribable to the different diet between the two groups: farmed specimens during farming (for about five months) were fed with a diet poor in Hg, such as Pacific mackerel *Scomber japonicus*, Atlantic mackerel *Scomber scombrus*, and Atlantic herring *Clupea harengus* that show Hg levels <0.05 mg/kg [37]. Therefore, farmed tuna in this period have lower Hg diet intake compared with wild ones that hunt in the Mediterranean Sea, a basin rich in cinnabar [38], which exhibits a high concentration of this toxic element. In fact, ABFT resident in the Mediterranean Sea show Hg concentrations 3–4 fold higher than ABFT that reach this site only during the spawning period [39]. No differences in Hg content in seawater is reported in the literature for the Mediterranean area [10], so the differences between groups are probably not due to seawater Hg levels. However, other factors could contribute to these results: the second point, strictly related to diet, is represented by the lipid content. In fact, farmed tuna have reduced mobility during farming and a diet rich in lipid content, in order to achieve in a few months, a weight 40% higher than the original one, and with a high amount of accumulated lipids for the purpose of selling a good and appreciated food to the consumer. This fact is evident on observing the results obtained for the lipid content, 12 ± 4% in farmed vs. 1.44 ± 1.47% in wild ones. A positive effect on Hg content in muscle is probably ascribable to lipids: in fact, Hg shows a high affinity with thiol group complexes within muscular tissue, and so it tends to be found associated with the protein fraction of tissues. When lipid content increases in muscle, such as during the farming period, a dilution effect on mercury in muscle tissue appears [25], irrespective of other variables (fish length, weight, etc.). Therefore, some authors [25,26,40] have observed a negative correlation between Hg and lipid percentage in tuna muscle tissue that could explain the results found in our research.

### 4.2. Selenium in Farmed and Wild Atlantic BlueFin Tuna (ABFT)

The analysis of Se in farmed and wild tuna shows twice the amount of Se in the first ones (farmed: 1.1 ± 0.9 mg/kg w.w. vs. wild: 0.6 ± 0.3 mg/kg w.w.) with a statistically significant difference (*t* = 2.60364, Df = 68, *p* = 0.0113, *p* < 0.05). This higher value of Se in farmed tuna compared to wild ones, could help reduce Hg bioavailability in this group, since Se binds to this element reducing its possibility to cause harm. hence. Mercury binds to selenium forming insoluble mercury selenides at the molecular level: this complex implicates less bioavailability of Hg and, consequently, less toxicity [41,42]. This effect on Hg bioavailability is evident in trophic transfer which for apical predators such as tuna is very important. Diet could represent a key role in this equilibrium since a diet rich in Se could reduce the risk for consuming seafood containing high levels of bioavailable Hg. In our study, the farmed fish diet (based on Pacific mackerel *Scomber japonicus*, Atlantic mackerel *Scomber scombrus*, and Atlantic herring *Clupea harengus*) should have good levels of Se ranging from 0.5–08 mg/kg [43,44,45,46,47] in order to provide tuna with a suitable dose of this element.

### 4.3. Risk Assessment: Selenium vs. Mercury Indices

**Figure 4:**
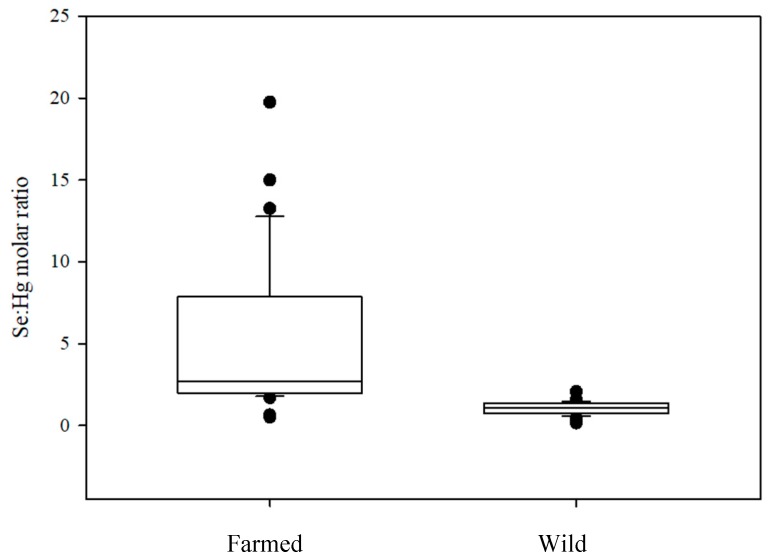
Boxplots of Se:Hg molar ratio in farmed and wild tuna.

Selenium/Hg molar ratio has been generally used for the evaluation of risks posed by Hg exposure from seafood consumption and when this ratio approaches or exceeds 1, the food could be deemed safe for consumers. Our results (Figure 4) show that Se:Hg ratio is higher in farmed tuna (5.10 ± 0.38) compared to wild ones (0.96 ± 0.06), underlining the benefit of the former seafood for consumers. 

**Figure 5:**
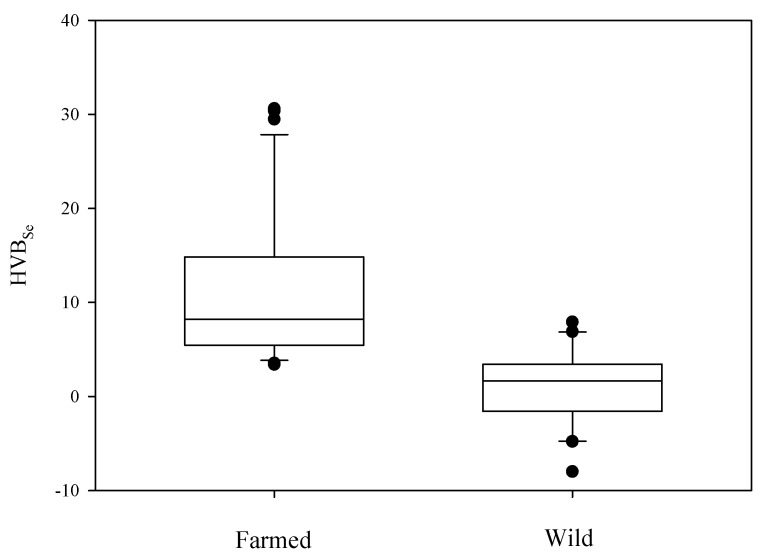
Boxplots of HBVSe molar ratio in farmed and wild tuna.

No significant differences between male and female were found for both groups, whereas differences in the relationship with size were found. In fact, farmed tuna show no trend with weight of specimens unlike wild groups where Se:Hg decreases on increasing the body weight of fish, as expected, since Hg content in wild tuna increases with increasing size. However, recently a new parameter, the selenium health benefit value (HBVSe), was introduced to better understand the quote of bioavailable selenium that remains after its mercury-interaction [24,48] as explained in the materials and methods section. In this case, a positive sign of HBV_Se_ would negate risks associated with Hg exposure, whereas negative values are associated with seafood potentially dangerous for Hg toxicity. Relative to this index, farmed tuna seems to be an excellent seafood, showing a HBV_Se_ of 11.16 vs. 0.29 for wild ones (Figure 5). Even for this index, no differences between sexes are reported and a negative correlation between HBV_Se_ and weight was found for wild ones. So farmed tuna is considered a healthy food compared with wild specimens, often preferred by consumers.

### 4.4. Comparison with Law Limit

The Commission Regulation in force on Hg concentration in seafood (EC No 1881/2006 of 19 December 2006 setting maximum levels for certain contaminants in foodstuffs) has fixed Hg maximum levels in tuna species (*Thunnus species*, *Euthynnus species*, *Katsuwonus pelamis*) at 1 mg/kg (w.w). Our results demonstrate that almost all farmed tuna (93%) show Hg levels below the established limit, whereas for wild ones only 9% are deemed safe according to mercury levels. Regarding with Se, no regulation exists for food/seafood, since it is an essential element and so it is not possible to make comparisons with the legal limit. However, the recommended dietary allowance (RDA) for Se [49] (based on the amount needed to maximize synthesis of the selenoprotein glutathione peroxidase) is 55 µg (0.7 µmol)/day whereas the tolerable upper intake level (UL) for adults is set at 400 µg (5.1 µmol)/day based on selenosis as the adverse effect. Therefore, considering a portion of 150 g tuna per meal, farmed tuna supplies 160.5 µg of Se content daily whereas wild specimens only 96 µg. Even if wild tuna provides a right quote of Se, farmed ones could be suitable for major apportionment of this element.

### 4.5. Comparison with Literature Data

Table 3 summarizes the literature data on Hg and Se in tuna sampled in the Mediterranean or other sea/ocean in the world. Few studies have been carried out in the Mediterranean Sea even though it is well known that tuna, being a top predator, can readily accumulate contaminants. Generally, the levels of Hg and Se found in our study are well consistent with the reported data, although the differences in size/weight make the comparisons less precise. Concerning mercury, farmed tuna show similar levels compared with the same species and same area: all farmed reports show Hg values well below the legal limit [20,50,51]. For wild *Thunnus thynnus,* our study confirms that Mediterranean tuna have Hg levels higher than tuna coming from other areas such as Australia or Japan [20,40], even compared to other tuna species, such as *T. albacares, T. alalunga, T. obesus* that usually spend their time life principally in the Atlantic, Pacific and Indian oceans, coming rarely into the Mediterranean Sea, known as a Hg-rich basin [52]. Therefore, the Mediterranean environment seems to be crucial to explain these differences. Regarding Se, farmed tuna show the highest concentrations compared with other data (available only for wild specimens), except for Pacific and Atlantic Oceans [24,53]. Selenium levels in fish varies normally between 0.2–0.9 mg/kg [48] with good concentrations reported in apical species such as tuna and swordfish [54,55]. Among the top predators, Burger and Gochfeld [56] identified *T. thynnus* and *T. albacares* as the best species for Se content. In our study, since the only difference is represented by diet, a possible explanation for the higher value in farmed tuna is linked to this matter: in fact, during the farming period, the Se supply could be richer than in the wild diet where the preys are very variable in type and hence in selenium content. A future study on Hg and Se levels in the diet of farmed tuna will be planned to confirm these hypotheses.

Concerning risk assessment, our results on Se:Hg molar ratio are the lowest for wild tuna that showed a value of 1.32: no other results less than 3 are reported in Table 3. Even if some authors have stated that a Se:Hg value >1 gives protection against mercury [57], it is reasonable to assume that a value closer to the unit could be insufficient for essential selenium functions because all Se taken up through diet is involved in Hg sequestration, so wild *Thunnus thynnus* does not appear to represent a suitable seafood for consumers, since the value is very close to unitary. Table 3 shows the great variability in Se:Hg ratio among pelagic fish, with values ranging from 1.3 (our study) to 14.12 (*T. albacares* [48]): yellowfin tuna are generally of smaller size that ABFT and this feature could represent the key of interpretation for this difference, as described by Polak-Juszczak [58], where a negative correlation between Se:Hg ratio and fish size was found.

An overview on HBV_Se_ is presented in Table 3, even if comparisons are limited since not much data are available in the literature and no studies on this index have been carried out in the Mediterranean Sea. However, the farmed tuna analysed in this study show similar values to different species of tuna sampled in other oceans/sea [24,48] where higher HBV_Se_ has been found in small size species (*T. albacares, T alalunga, T obesus*), caught out of the Mediterranean Sea. On the other hand, wild tuna analysed in this study shows lower HBV_Se_ than farmed one (values ranging from −59.39 to 10.7), attesting the unadvisable supply of this seafood for this index.

## 5. Conclusions

*Thunnus thynnus* represents a seafood product of high nutritional value and high commercial interest, especially in the last few years, with the rapid growth in the sushi market and the increasing awareness that fish consumption is healthy. However, contamination of seafood is a well-recognized question for top predators such as tuna, especially with reference to toxic metals like mercury, and in particular for fish sampled in the Mediterranean area, a basin rich in this element. For this purpose, we evaluated the levels of Hg in *Thunnus thynnus* specimens, in the Mediterranean Sea, both farmed and wild, to evaluate the bioaccumulation of this metal and the counteracting balancing effect of selenium.

This study, the first on tuna from the Mediterranean Sea, demonstrates that for mercury, farmed Atlantic bluefin tuna has a minor risk/benefit ratio and is safer than wild tuna. Mercury bioaccumulation, common in top predators from the Mediterranean Sea, could be strongly reduced by diet, providing foods from other seas, poor in Hg and rich in Se, to ensure safe seafood with levels of selenium satisfactory for body functions. Therefore, direct and indirect effects contribute to reducing mercury accumulation in tuna during the farming period. However, the limited availability of scientific data on this topic suggests that other studies will be useful to validate the role of diet on Hg level in muscle tissue. So, future studies on nutritional value of farmed fish—even in the framework of the Marine Strategy that aims to a good environmental status both for Descriptor 3 (the population of commercial fish species is healthy) and Descriptor 9 (contaminants in seafood are below safe levels)—are encouraged by these results, even to supply an ever growing request of tuna *Thunnus thynnus* on our dishes.

## Figures and Tables

**Table 1:** Biometric measurements of farmed Atlantic bluefin tuna (ABFT).

	Sample	Weight, kg	Length, cm
Male	2	290	243
	4	327	256
	5	211	233
	8	258	247
	13	289	244
	14	216	222
	15	93	177
	24	122	186
	30	408	270
	31	298	247
	34	294	247
	35	317	252
	36	379	269
	37	308	253
	38	287	246
	40	306	255
	41	355	260
	42	334	255
Female	1	305	244
	3	268	252
	7	168	217
	9	225	228
	10	190	223
	11	188	218
	12	160	210
	17	323	255
	18	183	220
	19	220	225
	20	54	154
	21	57	149
	22	89	169
	23	88	180
	25	233	227
	27	159	204
	28	195	228
	29	227	229
	33	320	247
	39	302	250

**Table 2:** Biometric measurements of wild ABFT.

	Sample	Weight, kg	Length, cm
Male	89	32.3	122
	90	42.1	129
	91	57.4	152
	92	46.4	138
	93	51.9	142
	95	29	111
	96	44.8	136
	97	34.2	125
	204	48.3	135
	206	38.1	121
	208	22.3	110
	210	56.2	143
	212	45.7	128
	213	47.1	128
	215	38.8	122
	357	27.5	114
	361	50.7	140
	365	27.5	117
Female	88	37.1	124
	94	49.1	118
	203	51.9	146
	209	39	126
	216	47.4	133
	354	39	130126
	355	35.9	125
	356	30.9	121
	360	45.7	140
	362	45.2	135
	363	56.2	147
	364	48.3	138
	366	32	125

**Table 3:** Comparison with literature data.

Sampling Area	Length (cm) or Weight (kg)	Hg Tot. (mg/kg ww) M ± DS (Min-Max)	Se tot. (mg/kg ww) M ± DS (Min-Max)	Se:Hg Molar Ratio	HBV_Se_ M ± DS (Min-Max)	References
*T. thynnus*:						
Malta (*farmed)*	238 ± 93 (W)	0.61 ± 0.20	1.07 ± 0.86	5.48	(−7.69–46.88)	This study
Sardinia	45 ± 26 (W)	1.68 ± 0.58	0.64 ± 0.31	1.32	(−59.39–10.7)	This study
Ionian Sea (*farmed*)	8–540 (W)	(0.28-1.28)				[51]
Australia	16.2–41.6 (W)	0.32 ± 0.03				[37]
Mediterranea Sea (*farmed*)	-	0.54–0.67				[50]
Japan	-	0.33–0.61				[20]
Strait of Messina	50–190 (W)	(2.45–4.21)				[59]
Ionian Sea	2.85–4.36 (W)	(0.13–0.35)				[60]
Castellon (ES)	0.7–1.085 (W)	(0.081–0.306)				[61]
Adriatic Sea (*farmed*)	100–300 (W)	(0.490–1.809)				[51]
Canary Islands (ES)	-	(0.298–0.779)				[54]
New Jersey (USA)	-	0.52 ± 0.034	0.43 ± 0.038	2.07	4.15	[62]
Mediterranean Sea	130–290 (W)	(0.246–0.714)	(0.27–1.21)			[63]
Tyrrenian Sea	0.33–158 (W)	(0.07–4.26)				[64]
Atlantic Ocean	>50 (W)	(0.24–0.90)	(0.58–2.3)			[53]
Mediterranean Sea	5.3–83 (W)	(0.16–2.59)				[65]
*T. alalunga*:						
Atlantic Ocean	-	(0.118–0.564)				[66]
Indian Ocean	5.3–83 (W)	0.478 ± 0.14				[67]
Central Pacific Ocean	22.6 ± 3.8 (L)	0.50 ± 0.24	0.88 ± 0.19	5.26	10.4	[16,48]
Mediterranea Sea	4.0–8.7 (W)	1.17 ± 0.23				[65]
*T. albacares*:						
South Africa (Atlantic Ocean)	29–50.8 (W)	0.80 ± 0.25				[68]
Atlantic Ocean	-	(0.166–0.531)				[66]
New Jersey (USA)	-	0.20 ± 0.025	0.47 ± 0.027	6.11		[62]
New Jersey (USA)	-	0.43 ± 0.05	0.62 ± 0.03	3.6		[69]
Indian Ocean	113 ± 13 (L)	0.38 ± 0.17				[67]
Pacific Ocean	41.1 ± 16.7 (L)	0.30 ± 0.18	1.25 ± 0.27	14.12	15.6	[24,48]
Mexico	60.5–94.2 (L)			10.29	6.55	[70]
Atlantic Ocean	-	(0.22–1.3)	(0.87–1.8)			[53]
*T. obesus*:						
Atlantic Ocean	-	(0.344–1.29)				[66]
Indian Ocean	87 ± 46 (L)	0.339 ± 0.29				[69]
Pacific Ocean	41.2 ± 20.4 (L)	0.60 ± 0.25	0.99 ± 0.28	5.17	10.0	[24,48]
*Thunnus***spp.**:						
Portugal market	-	0.31 ± 0.01	0.92 ± 0.01	8		[9,55]
Portugal market	-	0.37 ± 0.02	0.72 ± 0.01	4.9		[9]

## Supplementary Materials

The following are available online at https://www.mdpi.com/1420-3049/24/7/1273/s1, **(1) Texts**: Mercury and Selenium analysis, Laboratory and apparatus, Chemicals and Reagents; **(2)** Tables: Table S1. Parameter of microwave assisted digestion for tuna tissues, Table S2. Electrothermal program, Table S3. Mercury and selenium (mg/kg w.w.) concentrations in farmed ABFT, Table S4. Mercury and selenium (mg/kg w.w.) concentrations in wild ABFT.
