# Supplementary material for: Determination of Hg in Farmed and Wild Atlantic Bluefin Tuna (Thunnus thynnus L.) Muscle
Source: Molecules. 2019 Apr 1;24(7):1273. doi: 10.3390/molecules24071273 (PMC6480458; doi:10.3390/molecules24071273)
Supplement: Supplementary file 1 [file molecules-24-01273-s001.pdf]

Supplementary Material

Determination of Hg in Farmed and Wild Atlantic Bluefin Tuna (*Thunnus thynnus* L.) Muscle

Anna Annibaldi [1,*], Cristina Truzzi [1,*], Oliana Carnevali [1], Paolo Pignalosa [2], Martina Api [1], Giuseppe Scarponi [1] and Silvia Illuminati [1]

[1] Dipartimento di Scienze della Vita e dell'Ambiente, Università Politecnica delle Marche, Via Brecce Bianche, 60131 Ancona, Italy; o.carnevali@univpm.it (O.C.); m.api@pm.univpm.it (M.A.); g.scarponi@univpm.it (G.S.); s.illuminati@univpm.it (S.I.)

[2] Oceansis s.r.l., Ercolano NA 80056, Italy; oceanissrl@gmail.com

* Correspondence: a.annibaldi@univpm.it (A.A.); c.truzzi@univpm.it (C.T.)

Sample treatment

Table S1. Parameter of microwave assisted digestion for tuna tissues.

Step	Oven Power (W)	Power (%)	Time (min)	Pressure (psi)	Temperature (°C)	Hold time (min)
1	800	100	10	50	150	5
2	800	100	10	90	160	5
3	800	100	10	150	175	5

Mercury and Selenium analysis

The instrumental parameters were: wavelength 196 nm, slit 1 nm, lamp current 13 mA, background correction Zeeman effect, calibration mode peak height and the electrothermal program is reported in Table S2. A palladium solution (1 g/L, 10% nitric acid, 5% citric acid) was added as chemical matrix modifier and the standard addition technique applied for resolution of matrix effects.

Table S2. Electrothermal program

Program step	Temperature (°C)	Time (s)	Gas Flow (L/min)
1	85	5	0.3
2	95	40	0.3
3	120	10	0.3
4	1000	5	0.3
5	1000	1	0.3
6	1000	2	0
7	2600	0.8	0
8	2600	2	0
9	2600	2	0.3

Laboratory and apparatus

The laboratory analytical balance was the AT261 Mettler Toledo (Greifensee, Switzerland, readability 0.01 mg, repeatability SD = 0.015 mg). Variable volume micropipettes and neutral tips were from Brand (Wertheim, Germany, Transferpette). Scalpels with sterile stainless steel blades were from Granton (Mod. 91021, Sheffield, UK). The plastic containers used for sample storage were: low density polyethylene 30 mL cylindrical containers (Kartell, Milan, Italy, Mod K912), previously decontaminated as described elsewhere.

Chemicals and Reagents

Ultrapure water was obtained from a two-stage system Midi (Elix and Milli-Q) from Millipore (Bedford, MA, USA). The dogfish muscle certified reference material (CRM) DORM-2 was obtained from the National Research Council of Canada. Atomic absorption standard solutions of inorganic selenium, palladium and mercury (Carlo Erba Milan, Italy, 1.0 g/L) were used for the relative analysis. All stock standard solutions were stored in a refrigerator at 4 °C and protected from light. Working standard solutions were prepared just before use by appropriate dilution of the stock standard solution. Citric acid powder was purchased from Sigma Aldrich. Superpure nitric acid (67–69%) and hydrogen peroxide (30%) were purchased from Carlo Erba

Results

All results are expressed in wet weight (w.w).

Table S3. Mercury and selenium (mg/kg w.w.) concentrations in farmed ABFT.

	Sample	Hg, mg/kg	Se, mg/kg
Male	2	0.55±0.01	0.52±0.03
	4	0.62±0.03	-
	5	0.48±0.01	2.41±0.11
	8	0.58±0.002	2.35±0.38
	13	0.86±0.03	0.63±0.08
	14	0.41±0.03	0.380±0.005
	15	0.69±0.03	0.78±0.01
	24	0.60±0.03	0.54±0.05
	30	1.05±0.90	0.76±0.04
	31	0.85±0.05	0.89±0.11
	34	0.51±0.04	2.43±0.02
	35	0.47±0.001	0.42±0.004
	36	1.11±0.02	0.23±0.04
	37	0.47±0.03	1.07±0.03
	38	0.70±0.003	0.71±0.05
	40	0.48±0.03	3.71±0.02
	41	0.37±0.01	2.18±0.03
	42	0.60±0.003	2.07±0.02
Female	1	1.14±0.07	0.29±0.005
	3	0.70±0.02	0.63±0.06
	7	0.49±0.01	1.28±0.09
	9	0.70±0.003	0.59±0.01
	10	0.40±0.02	0.96±0.005
	11	0.43±0.02	0.49±0.004
	12	0.64±0.01	3.32±0.03
	17	0.58±0.02	0.55±0.01

Table S3. *Cont.*

	18	0.41±0.02	0.95±0.06
	19	0.52±0.01	1.70±0.12
	20	0.86±0.02	0.93±0.01
	21	0.85±0.02	0.61±0.01
	22	0.52±0.01	0.39±0.01
	23	0.61±0.02	0.41±0.01
	25	0.58±0.02	0.44±0.01
	27	0.50±0.001	0.38±0.01
	28	0.43±0.03	1.32±0.04
	29	0.47±0.02	1.47±0.13
	33	0.52±0.02	0.77±0.01
	39	0.58±0.01	0.46±0.01

Table S4. Mercury and selenium (mg/kg w.w.) concentrations in wild ABFT

	**Sample**	**Hg, mg/kg**	**Se, mg/kg**
Male	89	0.97±0.17	0.40±0.02
	90	1.79±0.19	0.31±0.02
	91	1.04±0.07	0.56±0.03
	92	2.19±0.11	0.86±0.01
	93	2.47±0.25	1.04±0.01
	95	1.85±0.18	1.05±0.02
	96	1.59±0.11	0.86±0.03
	97	1.73±0.10	0.99±0.08
	204	1.61±0.06	0.64±0.02
	206	1.21±0.05	0.32±0.02
	208	0.76±0.03	0.27±0.03
	210	2.26±0.15	1.41±0.05
	212	1.45±0.06	0.41±0.03
	213	1.48±0.11	0.52±0.02
	215	1.86±0.06	0.67±0.002
	357	1.15±0.03	0.94±0.08
	361	1.51±0.10	0.59±0.02
	365	0.79±0.02	0.44±0.01
Female	88	1.05±0.05	0.51±0.01
	94	1.82±0.17	0.43±0.07
	203	1.35±0.05	0.60±0.01
	209	1.60±0.11	0.80±0.01
	216	2.03±0.11	0.66±0.04
	354	2.10±0.06	0.14±0.01
	355	-	0.14±0.01
	356	-	0.25±0.01
	360	2.39±0.13	0.64±0.02
	362	2.05±0.09	1.18±0.01
	363	3.39±0.10	0.77±0.04
	364	2.14±0.22	0.59±0.01
	366	1.12±0.07	0.56±0.01
